# Combination of Iodine-125 brachytherapy and chemotherapy for locally recurrent stage III non-small cell lung cancer after concurrent chemoradiotherapy

**DOI:** 10.1186/s12885-015-1657-3

**Published:** 2015-10-06

**Authors:** Xiaojuan Yu, Jin Li, Xiaoming Zhong, Jingdong He

**Affiliations:** Department of Oncology, Huai’an First People’s Hospital, Nanjing Medical University, 6 Beijing Road West, Huai’an, Jiangsu 223300 China

**Keywords:** Brachytherapy, Iodine-125, Non-small cell lung cancer, Recurrence

## Abstract

**Background:**

Locally recurrent non-small cell lung cancer (NSCLC) poses a great challenge to physicians. This study aimed to explore the efficacy and safety of the combination of brachytherapy and docetaxel and cisplatin for the treatment of locally recurrent stage III NSCLC.

**Methods:**

Fifty two patients with locally recurrent stage III NSCLC after concurrent chemoradiotherapy were randomly divided into two groups (*n* = 26). The patients in experimental group were treated with implantation of radioactive ^125^I seeds and DP regimen (docetaxel 60 mg/m^2^/cisplatin 75 mg/m^2^). Patients in control group received DP chemotherapy. The local control rate (LCR), progression-free survival (PFS), and overall response rate (ORR) were defined according to the Response Evaluation Criteria in Solid Tumors (RECIST).

**Results:**

With a median follow-up time of 11 months, PFS and LCR was 8 months (95 % CI: 6.99–9.01 months) vs. 5.5 months (95 % CI: 4.43–6.57 months) (*P* < 0.05) and 10 months (95 % CI: 8.72–11.28 months) vs. 6.2 months (95 % CI: 5.27–7.13 months) (*P* < 0.05) in the experimental and control groups, respectively. The ORR did not differ between treatment groups and was noted to be 69.2 % and 57.7 %, respectively (*P* >0.05). There was no occurrence of severe complications in experimental and control groups.

**Conclusion:**

The combination of ^125^I brachytherapy and second-line chemotherapy is superior to chemotherapy alone and is an effective and safe therapy for this disease.

**Trial registration number:**

ChiCTR-IOR-15006560

## Background

Lung cancer is the leading cause of malignant tumor death. Non-small cell lung cancer (NSCLC) accounts for 80–85 % of lung cancers. For advanced inoperable NSCLC, combined chemoradiotherapy is considered as the standard first-line treatment [1]. Phase III clinical trials have demonstrated that docetaxel is an effective therapeutic agent for recurrent NSCLC after first-line treatment. Nevertheless, for the patients who are treated with docetaxel-based second-line therapy, their overall survival (OS) is usually short and prognosis is poor [2].

Treatment of locally recurrent NSCLC after first-line concurrent chemoradiotherapy poses a great challenge to physicians [3]. Although chemoradiotherapy can be considered as an option for subsequent treatment, its application is strictly limited due to the normal tissue tolerance dose of radiation and the development of severe complications, including chronic basic pulmonary diseases (e.g. chronic obstructive emphysema), radiation pneumonitis, radiation esophagitis, and tracheal necrosis [4].

Currently, implantation of iodine-125 (^125^I) seeds is a treatment option for interstitial brachytherapy in lung cancer [5]. The implanted ^125^I seeds can generate a high dose (140–160 Gy) within target tumor volumes to continuously destroy tumor cells, while the surrounding non-neoplastic tissues only receive a very low dose and subject to little damage. In addition, the low radiation dose rate can induce the reoxygenation and increased blood flow of hypoxic tumor volumes, thus producing radiation-induced bystander effect to kill tumor cells that can overcome the inhomogeneous distribution of radiation dose [6, 7]. Percutaneous CT-guided ^125^I seed permanent implantation can decrease the local recurrence in the treatment of high-risk stage I NSCLC with less tissue injury and few complications [8]. ^125^I seed implantation decrease the local recurrence, especially in those with a positive cytology at the staple line [9]. Therefore, we hypothesized that the combination of ^125^I brachytherapy and second-line chemotherapy may be ideal for the treatment of locally recurrent stage III non-small cell lung cancer after first-line therapy. Nevertheless, this combined therapy is uncommonly used and its efficacy and safety remain unclear. In this study we performed a systematic assessment on the clinical efficacy of the combination of brachytherapy with radioactive iodine-125 (^125^I) seeds and chemotherapy for the treatment of locally recurrent stage III NSCLC.

## Methods

### Patient selection and inclusion

Inclusion criteria were as follows: patients with locally recurrent stage III NSCLC within one year after receiving concurrent chemoradiotherapy; time of survival was estimated to be longer than three months; tumor diameter was less than 6 cm; no severe liver insufficiency or renal insufficiency, heart diseases, diabetes, coagulation dysfunction, and other chronic diseases; no severer chronic obstructive pulmonary diseases; the Eastern Cooperative Oncology Group (ECOG) score was no more than 2; tumor lesion was suitable for brachytherapy, as the location and size of the lesion were confirmed by chest CT scanning; patients intended to receive the treatments; patients did not receive any radiotherapy and chemotherapy within 3 months of this study. Local recurrence was defined as a 20 % increase in the volume of primary tumor mass from the point of maximum tumor regression in the lung without lymph node metastasis. All patients were evaluated by the investigators of this study (including the oncologists, surgical specialists, and radiologists of Huai’an First People’s Hospital Affiliated to Nanjing Medical University) and confirmed to be able to receive the second-line chemotherapy by paclitaxel (135 mg/m^2^, at day 1) plus cisplatin (75 mg/m^2^, at day 2) and CT-guided radioactive ^125^I seed implantation.

Based on pathological conformation, 52 patients with locally recurrent lung cancer within 3–12 months after receiving concurrent chemoradiotherapy were included in this study. Prior to the procedure, all patients received multiple imaging examinations including chest CT, abdominal CT or magnatic resonance imaging (MRI), cranial MRI, and bone emission computed tomography (ECT), blood routine examination, blood coagulation tests, hepatic and renal function tests, pulmonary function tests, and electrocardiogram (ECG). Tumor staging was according to American Joint Committee on Cancer (AJCC) staging manual (7th edition). All patients were previously treated by 6- or 15-MV X-ray beams produced by Siemens ONCOR Expression Linear Accelerator (Siemens AG, Muenchen, Germany). The dose was 2 Gy once daily, 5 times a week, for 6–7 weeks. The total radiation dose received was 66–70 Gy. The concurrent chemotherapy was paclitaxel (135 mg/m^2^, at day 1) plus cisplatin (75 mg/m^2^, at day 2), which was intravenously infused prior to radiotherapy and repeated after four weeks, for four cycles. The median time to recurrence was 7.9 months (range: 5.5–11.2 months). The study protocols were approved by Ethics Committee of Huai’an First People’s Hospital Affiliated to Nanjing Medical University and all patients gave signed informed consent.

### Study design

This was a prospective study (Trial registration number ChiCTR-IOR-15006560). The 52 patients were divided into two groups using computer-produced digital random method: experimental group, the patients received the combined therapy of 125I seed implantation, docetaxel (60 mg/m2 at day 1), and cisplatin (75 mg/m2 at day 2); control group, the patients received the combination of docetaxel (60 mg/m2 at day 1) and cisplatin (75 mg/m2 at day 2). Chemotherapy cycle length was three weeks and planned duration of chemotherapy was four cycles. Primary endpoints were progression-free survival and time of local control. CT/PET scans were not available during the years of the study due to economic reasons.

### CT-guided implantation of ^125^I seeds

Sixty-four slice spiral CT scanner (SIEMENS Somatom Sensation 64 CT Scanner) was provided by Siemens. Radiotherapy treatment planning system (TPS) HGGR-2000 was provided by Zhuhai Hokai Medical Instruments Co., Ltd (Zhuhai, China). Implantation needle (18 Gauge), implantation gun, and ^125^I seeds were provided by Ningbo Jun’an Pharmaceutical Technology Co., Ltd (Ningbo, China). The planning target volume (PTV) of lung tumor was defined and outlined after CT scan. Radiotherapy treatment plan for each patient was optimized according to the safety margin around the tumor volume or normal tissues and the radiation dose and radioactivity of ^125^I seeds. The ^125^I radioactive seeds used in this study had a length of 4.5 mm and a diameter of 0.8 mm, with an average energy of 27–32 keV, a half life of 59.6 days, and a tissue penetration range of 1.7 cm. The initial dose rate was 7 cGy/h prescribed to 1 cm depth, and the prescription dose was 90–110 Gy. The ideal position for radioactive seed implantation was determined based on pre-operative TPS and physical condition of patients. The correct entry point, the direction of needle advancement, and the space between implanted ^125^I seeds were determined under CT-guidance. After intramuscular injection of 10 mg diazepam and 100 mg pethidine hydrochloride, ^125^I seeds were implanted in the tissues via needles. The space between the implanted seeds was 1.5 cm. The activity of ^125^I seeds was 2.22–2.59 x 10^7^ Bq. Post-operative chest CT examination was performed to monitor the implantation-related complications (e.g. pulmonary hemorrhage, pneumothorax, or migration of radioactive seeds). Acquired CT images were then transferred to TPS for the dosimetry evaluation of implanted ^125^I seeds. The evaluation indicators included the tumor matched peripheral dose (MPD) and the dose that 90 % of the target volume received (D_90_). Postoperative monitoring of vital signs was conducted for all patients. Antibiotic prophylaxis was used to prevent post-operative infection. One week after the implatation, hemogram was examined to detect any complications.

### Evaluation of efficacy and safety

The efficacy was evaluated three months after interstitial permanent implantation of ^125^I seeds. Tumor response was evaluated based on imaging findings in accordance with the Response Evaluation Criteria in Solid Tumors (RECIST) criteria: complete response (CR), complete disappearance of target lesions (negative findings or only funicular shadows on imaging evaluation); partial response (PR), at least a 30 % decrease in the sum of the longest diameters of target lesions; progressive disease (PD), at least a 20 % increase in the sum of the longest diameters of target lesions or the appearance of one or more new lesions; stable disease (SD), neither sufficient shrinkage to qualify for PR nor sufficient increase to qualify for PD. The response rate (RR) of treatment was calculated using the following formula: RR = (CR + PR)/total number of patients × 100 %. The acute and late radiation toxicities were assessed according to toxicity criteria of the Radiation Therapy Oncology Group (RTOG) and the European Organization for Research and Treatment of Cancer (EORTC) [10].

### Follow-up

All patients were followed up from January 2006 until the time of local recurrence and disease progression. The range of follow-up was 4.5–24 months, with a median of 11 months.

### Statistical analysis

Statistical analysis was performed using SPSS 13.0 software. Kaplan-Meier survival curve method was used to estimate local control rate and PFS. The log-rank test was used to compare the difference between two treatment groups. Fisher’s exact test was used to assess the difference in CR rate and short-term efficacy between both groups. *P* < 0.05 was considered statistically significant.

## Results

**Fig. 1:**
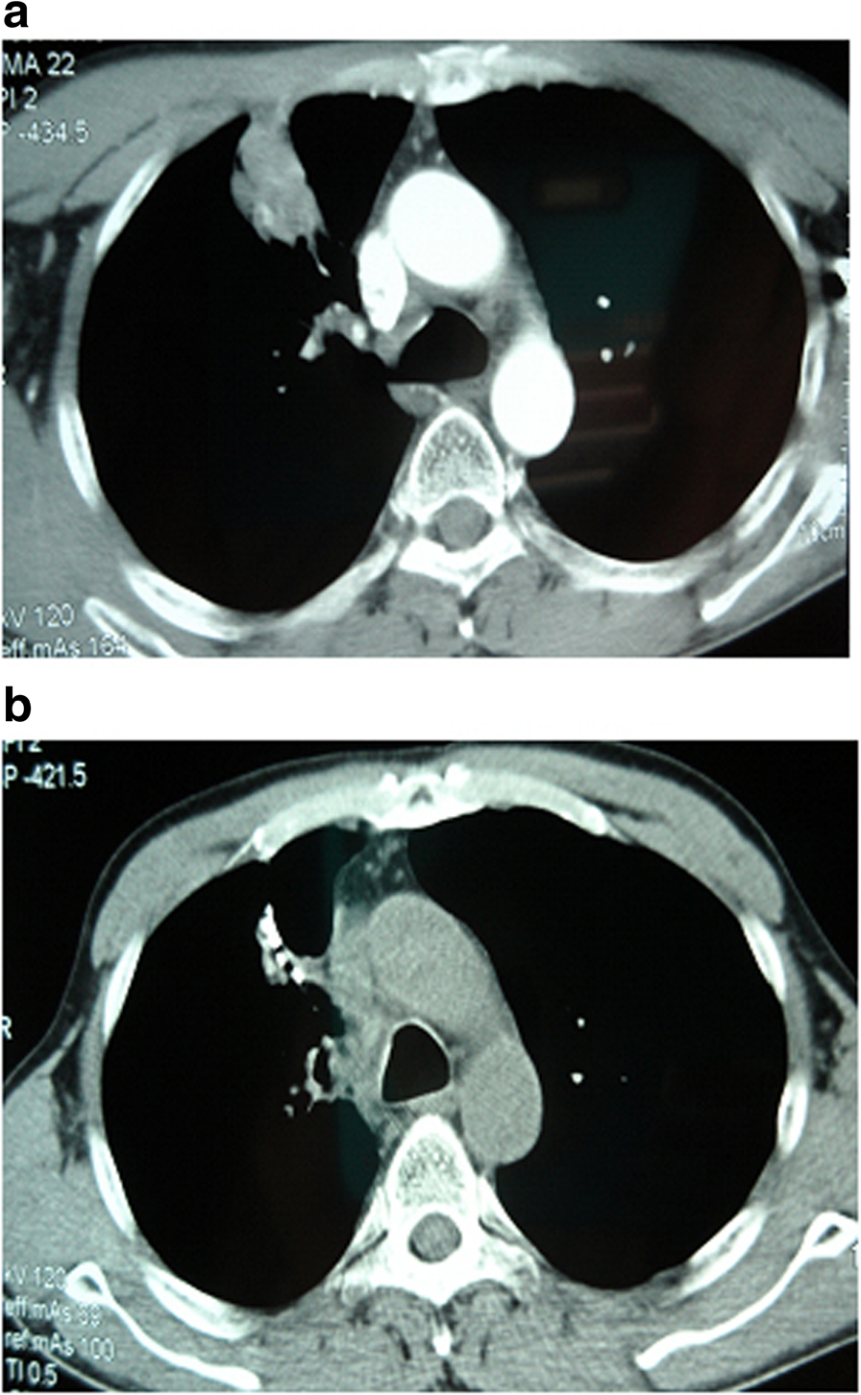
The reccurent tumot lesions in a representative patient who previsouly received concurrent chemoradiotherapy (**a**). After the combination of ^125^I brachytherapy and DP regimen, complete disappearance of target lesions was achieved (**b**)

Of 26 patients who received ^125^I brachytherapy, 15 were male and 11 were female. The median age was 62 years (age range: 48–72 years). Nine patients had squamous cell carcinoma, 16 had adenocarcinoma, and one had large cell carcinoma. Fourteen patients had stage IIIa NSCLC and 12 had stage IIIb NSCLC (Table 1). The number of implanted ^125^I seeds in these patients ranged between 15 and 92, with a median number of 34. The immediate post-operative CT scan demonstrated that the tumor MPD in these patients ranged between 90.2 and 130.6 Gy, with a median dose of 110.0 Gy. The D_90_ was 103.6–148.2 Gy in these patients, with a median dose of 128.9 Gy (Table 2). After brachytherapy, re-implantation of ^125^I seeds was required in one patient. Three patients had mild pneumothorax, two patients had hemoptysis, two had low-grade fever, and seed migration was observed in one patient. The patients with mild pneumothorax received conservative therapy and finally recovered completely. Another patient with grade III radiation pneumonitis obtained symptom relief after drug therapy (Table 3). There were no grade 4/5 complications in both experimental and control groups. The CT scan images of one representative patient were shown in Fig. 1.

**Table 1:** Baseline characteristics of patients enrolled in this study

Characteristics		Number of cases		P value
		Experimental group (*N* = 26)	Control group (*N* = 26)	
Gender	Male	15	17	0.776
	Female	11	9	
Stage	IIIA	14	16	0.779
	IIIB	12	10	
ECOG score	0–1	22	20	0.726
	2	4	6	
Pathology	Squamous cell carcinomas	9	11	0.656
	Adenocarcinomas	16	13	
	Large-cell carcinomas	1	2	
Differentiation	Well differentiated	7	3	0.291
	Poorly differentiated	19	23	
Tumor size (diameter)	3 cm	16	20	0.368
	3–6 cm	10	6

**Table 2:** Parameters of brachytherapy with radioactive ^125^I seeds

Parameters	Median value	Range
MPD (Gy)	110.0	90.2–130.6
D_90_ (Gy)	128.9	103.6–148.2
Number of seeds implanted	34	15–92
Activity (mCi per seed)	0.6	0.6–0.9
Dose rate (Gy/h)	0.07	0.05–0.09

**Table 3:** Complications in patients after brachytherapy with radioactive ^125^I seeds

Complications	Experimental group (*n* = 26)	Control group (*n* = 26)
Grade 4/5 complications	0	0
Mild complications		
Mild pneumothorax	3	3
Hemoptysi	2	3
Low-grade fever (38.5 C)	2	5
Radiation pneumonitis	1	0
Seed migration	1	0
Local skin erythema	0	1
No complications	17	14

For patients who received the combined therapy of ^125^I brachytherapy and DP chemotherapy, the time of local control was between 4.5 and 24 months, with a median of 10 months (95 % CI: 8.72–11.28 months). Of the patients in this cohort, three achieved progression free of disease after 24-month follow-up. The median PFS was 8 months (95 % CI: 6.99–9.01 months). The range of PFS was 3–24 months. Three months after ^125^I brachytherapy, the overall response rate (ORR) was 69.2 % (18/26). The rates of CR, PR, SD, and PD in this cohort were 38.5 % (10/26), 30.8 % (8/26), 23.8 % (6/26), and 7.7 % (2/26), respectively. For the control group, the time of local control was 3–15 months, with a median of 6.2 months (95 % CI: 5.27–7.13 months). The median PFS was 5.5 months (95 % CI: 4.43–6.57 months). The range of PFS was 3–10 months. The overall response rate was 57.7 % (15/26). The rates of CR, PR, SD, and PD in this cohort were 7.7 % (2/26), 50.0 % (13/26), 30.8 % (8/26), and 11.5 % (3/26), respectively.

**Fig. 2:**
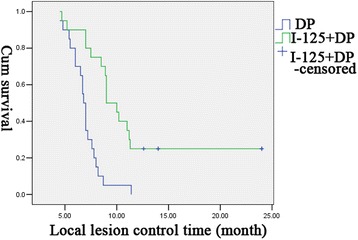
Comparasion of the time of local control in both treatment groups

**Fig. 3:**
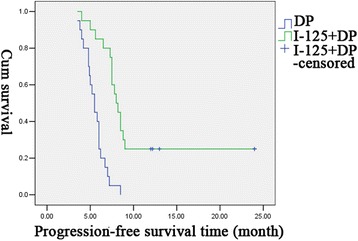
Comparasion of PFS in both treatment groups

There was no significant difference in ORR between the experimental group and the control group (*χ*2 = 0.75, *P* = 0.57 > 0.05). For the rate of CR, the time of local control, and PFS, there was significant difference between both groups (*χ*2 = 7.43, *P* = 0.02 < 0.05 for the rate of CR; *χ*2 = 8.59, *P* = 0.003 < 0.01 for the time of local control; *χ*2 = 4.7, *P* = 0.04 < 0.05 for PFS) (Figs. 2 and 3). The median time of local control in the experimental group and the control group was 10 and 6.2 months, respectively. There was significant difference in this outcome between two groups (*P* < 0.05). For PFS, the experimental group had a median of 8.0 months, which was significantly longer than that in the control group (5.5 months) (*P* < 0.05).

## Discussion

Lung cancer is the most common cancer worldwide. The majority of lung cancer patients are not diagnosed until the disease is at the relatively late stage. The patients with advanced lung cancer usually have a very short survival. Currently, lung cancer therapies are not satisfactory in their efficacy of improving the survival of patients. Clinical studies have proven that concurrent chemoradiotherapy is a standard treatment for locally advanced NSCLC, which can provide a 2-year survival rate of 39.7 %, a median OS of up to 22 months, and a median PFS of 17 months [11]. However, for the patients with locally recurrent stage III NSCLC (stage IIIA and III B) after concurrent chemoradiotherapy, the treatment mainly depends on second-line chemotherapy or molecular targeted therapy, which can provide a median OS of 11.7–12.2 months, a median PFS of 2–3 months, and a one-year survival rate of 30 % [12]. However, these therapies are not yet considered as the standard treatment for locally recurrent stage III NSCLC after concurrent chemoradiotherapy, further investigations are required to explore and optimize possible treatment regimens.

Development of computerized three-dimensional TPS has attracted more attention of physicians on CT-guide brachytherapy with radioactive seeds for treating malignant tumors [13]. The ^125^I radioactive seeds have an effective emission range of 1.7 cm in tissue. During the half-life of ^125^I radioactive seeds (59.6 days), tumor cells at different phases of the cell cycle can be destroyed by the gamma-rays emitted from the radioactive seed. Previous studies have shown that the low radiation dose rate could increase the sensitivity of hypoxic tumor cells to radioactive rays and produce radiation-induced bystander effect to kill tumor cells [7, 14]. In vitro studies have shown that ^125^I radioactive seeds up-regulated apoptosis-related genes, and regulated cell cycle and apoptosis of tumor cells [15]. Taken together, ^125^I radioactive seeds have the advantages including high conformality index and producing high dose in target volumes and low radioactive exposure to surrounding normal tissues, thus having great potential in the treatment of locally recurrent tumors [16].

Currently, brachytherapy with ^125^I seeds are widely used in clinic for the treatment of recurrent and metastatic prostate tumor and head and neck tumors [17–19]. However, this therapy has been rarely reported for treating patients with locally recurrent NSCLC. In this study we investigated the clinical efficacy of the combination of ^125^I brachytherapy and chemotherapy in the patients with locally recurrent stage III NSCLC after concurrent chemoradiotherapy. Our results demonstrated that this combined therapy achieved satisfactory efficacy compared with chemotherapy alone. For the combined therapy, the median time of local control was 10 months. Three patients achieved progression free of disease after 24-month follow-up. The median PFS was 8 months. Three months after ^125^I brachytherapy, ORR was 69.2 % (18/26). The rates of CR and PR were 38.5 % (10/26) and 30.8 % (8/26), respectively, which were significantly higher than those in the control group. Recent studies have indicated that local disease control rate is an important independent prognostic factor for the OS of patients with locally advanced lung cancer [20]. In the present study, ^125^I radioactive seeds were shown to have a good local tumor control. For three patients with well differentiated adenocarcinoma (tumor diameter < 3 cm with single metastatic lesion), the time of local control was longer than two years, suggesting that the degree of tumor differentiation, growth rate, and tumor size could affect the local efficacy in NSCLC patients.

Furthermiore, there was no occurrence of severe complications during the study, indicating that combined therapy of ^125^I brachytherapy and DP regimen is effective and safe for the treatment of locally recurrent stage III NSCLC after concurrent chemoradiotherapy. Further large-sale studies are needed to verify the potential of the combination of ^125^I brachytherapy and second-line chemotherapy for treament of locally recurrent stage III NSCLC.

## Conclusions

The combination of ^125^I brachytherapy and second-line chemotherapy is superior to chemotherapy alone and is an effective and safe therapy for this disease.
